# Tensor decomposition of transportation temporal and spatial big data: A brief review

**DOI:** 10.1016/j.fmre.2024.03.032

**Published:** 2024-09-25

**Authors:** Linchao Li, Xiang Lin, Bin Ran, Bowen Du

**Affiliations:** a National Key Laboratory of Green and Long-Life Road Engineering in Extreme Environment (Shenzhen), Shenzhen University, Shenzhen 518060, China; b Department of Civil and Environmental Engineering, University of Wisconsin, Madison 53706, United States; c State Key Laboratory of Software Development Environment, Beihang University, Beijing 100191, China; d Key Laboratory of Structural Health Monitoring and Control, Shijiazhuang Tiedao University, Shijiazhuang 050043, China

**Keywords:** Intelligent transportation system, Data mining, Machine Learning, Survey, Traffic prediction

## Abstract

Recent development in sensing and communication technologies has made the collection of a large amount of traffic data easy and transportation engineering has entered the big data era. The massive traffic data provides some good opportunities for Intelligent Transportation System (ITS), while some great challenges because of its characteristics of large value, variety, velocity, veracity, and volume. In recent few years, tensor decomposition has played an important role in traffic data analytic solutions and attached great interest from both academic and industrial areas. In this paper, the preliminary background and the implementation of tensor decomposition are presented. Then, some recent studies of tensor decomposition for traffic data imputation, traffic state prediction, and analysis of travel pattern are reviewed. Furthermore, advantages and disadvantages are discussed. Finally, remaining challenges of the application of tensor decomposition in transportation engineering are pointed out.

## 1 Introduction

The rapid urbanization in developing countries has resulted in the dramatic growth of vehicles and population in the big city and bring amounts of pressure on the transportation infrastructures. However, public infrastructure cannot keep pace with urban sprawl which causes massive traffic congestion problems in term of traffic safety, air pollution, and wasted energy. Currently, it becomes a common sense that intelligent transportation system (ITS) is effective ways to solve the above challenges. Nowadays, a large number of sensors have been installed on the infrastructures, vehicles, and travelers to monitoring the operation of the transportation system. The real-time multi-source heterogeneous temporal and spatial data will be transmitted to ITS by advanced communication technology like Wireless Fidelity and 5G. Most importantly, the ITS should have the ability to handle the massive real-time data and fully mine them for better decision making. As reported in previous studies, the ITS called IBM InfoSphere has the ability to process 120,000 position data points per second.

In the early stage of traffic data mining studies, traffic flow data were always been represented by a time series and all samples were organized a vector. Since then, powerful statistical methods or machine learning methods have been often applied for investigating the useful information. Currently, with the increasing of the data, organizing the data as a matrix to find the wanted information becomes more and more popular. The row of the matrix is for time mode and the column of the matrix is for space mode. As a result, the matrix can be analyzed by two-way signal processing methods including independent component analysis (ICA), principal component analysis (PCA), or their extensions. Also, the matrix sometimes can be seen as a picture which could be well handled by image recognition methods including convolutional neural networks (CNN), support vector regression, and etc.

However, the mostly applied computing methods for traffic research are oriented for vector or matrix. Consequently, in order to implement the above advanced methods, some high-dimensional temporal or spatial correlations like daily patterns or weekly patterns have to been concatenated or stacked in one mode for generating a matrix. For traffic flow data, such unfolding inevitably misses some potential existing correlations among the folded dimensions such as daily pattern and weekly pattern. The correlations can be of research interests. As a result, to appropriately analyze the correlations among multiple dimensions, the traffic data mining methods particularly for a high-dimensional datasets are critical to ITS. During recent years, organizing the traffic data as a multi-way array named as tensor has been widely attempted for traffic data preprocessing and analysis. For a tensor, tensor decomposition is an effective and efficient way to exploit the correlations among multiple dimensions of the tensor.

**Fig. 1:**
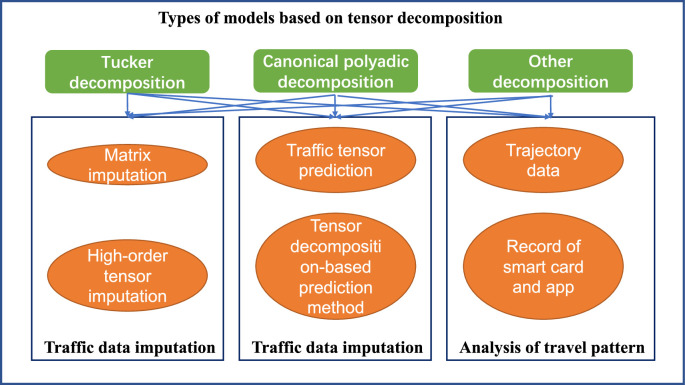
**Application of tensor decomposition in transportation engineering**.
• We carefully reviewed how various methods based on tensor decomposition have been applied in each application and present the evolving trend of the methods. Moreover, the tricks for design of the tensor and the procedures for decomposition are summarized. Based on the above presentations, it becomes easier for readers to know the state-of-the-art models in the corresponding application.
• The advantages, shortcomings, and applicability of tensor decomposition in ITS is concluded. Also, several future directions of tensor decomposition towards temporal and spatial traffic data are suggested in the paper for readers.

The tensor decomposition is firstly defined in mathematics field and has been popularized in the field of chemometrics and psychometrics for high-dimensional data analysis. Recently, tensor decomposition has attracted a great deal of attention for traffic data processing. In the past decades, there have been many review papers about tensor decomposition including its history, algorithms, and models. Also, some papers have reviewed the application of tensor decomposition in different fields, while there is no review specifically for tensor decomposition of traffic data yet. Therefore, this review paper aims to summarize previous studies concerned with tensor decomposition of traffic data and discussing some key issues regarding the future applications. Readers can be benefit from the review in the following ways:

• A wide coverage on the application of tensor decomposition has been presented for ITS. As shown in Fig. 1, tensor decomposition are mainly applied in three types of traffic data analysis tasks including traffic data imputation, traffic state prediction, and analysis of travel pattern. Also, we have addressed some other niche applications of tensor decomposition in ITS.

In the following parts of the paper, the preliminary background on tensor decomposition will be firstly introduced in Section 2. Subsequently, tensor decomposition applied for traffic data imputation, traffic state prediction, analysis of travel pattern, and some other tasks will be presented in 3, 4, 5, 6, respectively. Then, we will discuss the advantages, shortcomings, and applicability of tensor decomposition in ITS and address some suggestions about using tensor decomposition in Section 7. Finally, the paper will be concluded and some future directions are presented in Section 8.

## 2 Preliminary background on tensor decomposition

In this section, the preliminary background of tensor decomposition is presented. First, the notations and mathematical operations used in the paper are defined. Then, two widely used tensor decomposition called Canonical Polyadic (CP) and Tucker are introduced.

### 2.1 Notations and basic mathematical operations

The notations and basic mathematical operations used in this review are very similar to that in [[1], [2]].

#### 2.1.1 Notations

**Fig. 2:**
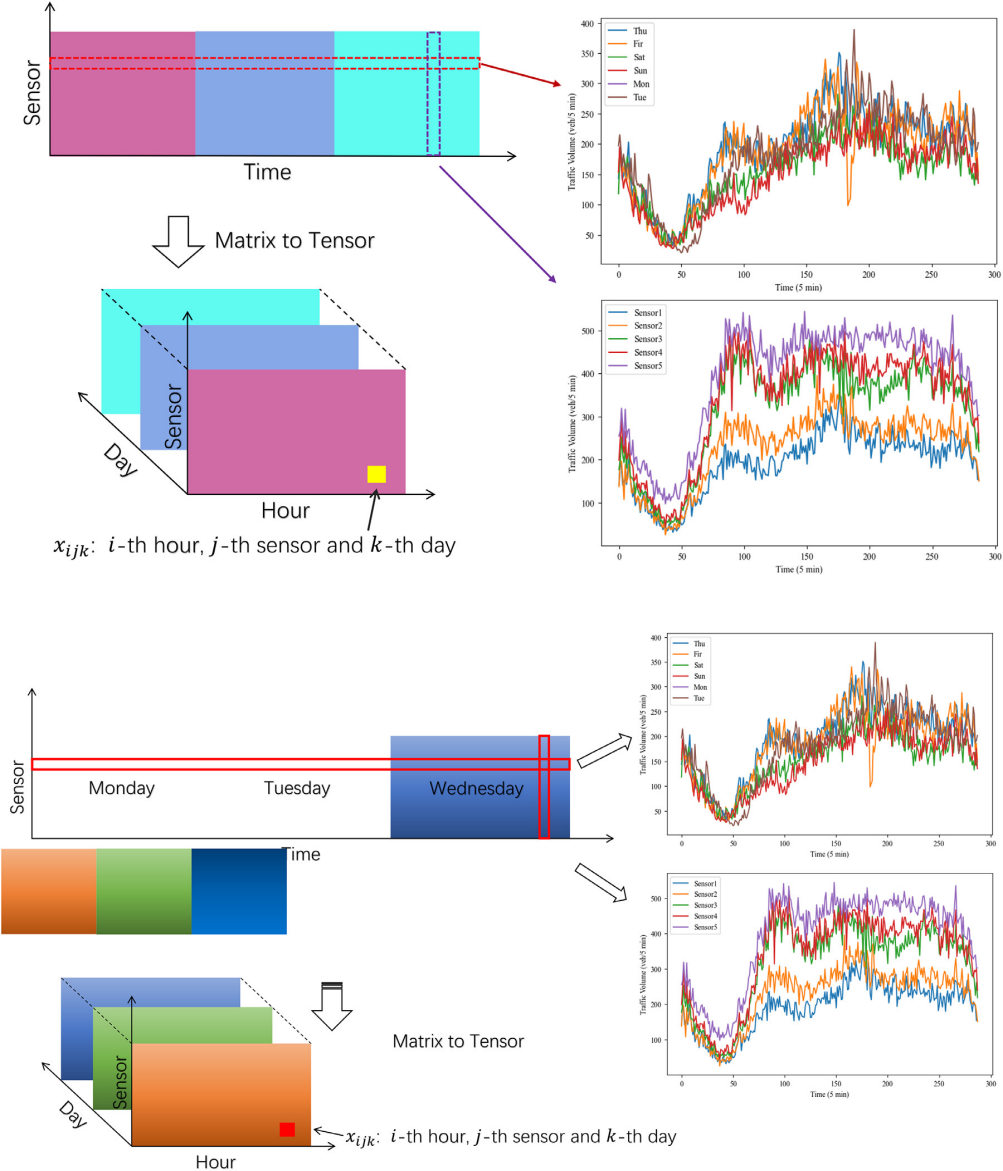
**An example of a three-order tensor (**A**) for traffic flow data**.

The number of dimensions of a tensor is called as order, ways, or modes for example a vector is a one-order tensor. Throughout this paper, scalars, vectors, matrices, and high-order tensors are denoted by lowercase letters, e.g., *a*, bold lowercase letters, e.g., **a**, bold capital letters, e.g., **A**, and bold Euler script letters, e.g., A, respectively. The *i*th value in a vector is denoted by $a_{i}$. The elements of a matrix and a three-order tensor are denoted by $a_{ij}$ and $a_{ijk}$, respectively. When every index is fixed except one, a fiber of a tensor can be obtained. Take a three-order tensor for example, $a_{:jk}$, $a_{i:k}$, and $a_{ij:}$ shown in Fig. 2 are called as column, row, and tube fibers, respectively. When all indices are fixed but two, a slice of a tensor can be obtained. Take a three-order tensor for example, $A_{i::}$, $A_{:j:}$, and $A_{::k}$ shown in Fig. 2 are called as horizontal, lateral, and frontal slides, respectively.

#### 2.1.2 Basic mathematical operations

• Matricization.
  All tensors can be reconstructed as matrices which is called as matricization, unfolding, or flattening. For a *N*-order tensor $A\in R^{I_{1}\times I_{2}\times \cdots \times I_{N}}$, it can be unfolded into *N* different matrices and the mode-*n* matricization is denoted by $A_{\left(n\right)}$. The element $\left(i_{1},i_{2},\ldots ,i_{N}\right)$ of the tensor equals to the element $\left(i_{n},j\right)$ of the mode-*n* matricization, where
  (1) j=1+∑k=1k≠nN(ik−1)Jk
  (2) Jk=∏m=1m≠nk−1Im
• Inner product
  The inner product of two tensors A and B having the same size is defined as
  (3) $$\langle A,B\rangle =\sum_{i_{1}=1}^{I_{1}}\sum_{i_{2}=1}^{I_{2}}\cdots \sum_{i_{N}=1}^{I_{N}}a_{i_{1}i_{2}\cdots i_{N}}b_{i_{1}i_{2}\cdots i_{N}}$$
• Outer product The outer product of vectors is represented by the symbolic ∘. A tensor is rank one if it can be written as
  (4) $$A=a^{\left(1\right)}\circ a^{\left(2\right)}\circ \cdots \circ a^{\left(N\right)}$$
  The element of the tensor can be calculated by
  (5) $$a_{i_{1}i_{2}\cdots i_{N}}=a_{i_{1}}^{\left(1\right)}a_{i_{2}}^{\left(2\right)}\cdots a_{i_{N}}^{\left(N\right)}\;\;1\leq i_{n}\leq I_{n}$$
• Kronecker product
  The Kronecker product of two matrices $A\in R^{I\times J}$ and $B\in R^{K\times L}$ is defined by
  (6) $$A⊗B=\left[\begin{array}{cccc} a_{11}B & a_{12}B & \cdots & a_{1J}B\\ a_{21}B & a_{22}B & \cdots & a_{2J}B\\ \vdots & \vdots & \ddots & \vdots \\ a_{I1}B & a_{I2}B & \cdots & a_{IJ}B \end{array}\right]$$
$=\left[\begin{array}{cccccc} a_{1}⊗b_{1} & a_{1}⊗b_{2} & a_{1}⊗b_{3} & \cdots & a_{J}⊗b_{L-1} & a_{J}⊗b_{L} \end{array}\right]$
• Khatri−−Rao product
  The Khatri−−Rao product of two matrices $A\in R^{I\times K}$ and $B\in R^{J\times K}$ having the same number of column is defined by
  (7) $$A⊙B=\left[\begin{array}{cccc} a_{1}⊗b_{1} & a_{2}⊗b_{2} & \cdots & a_{K}⊗b_{K} \end{array}\right]$$
• Hadamard product
  The Hadamard product of two matrices $A\in R^{I\times J}$ and $B\in R^{I\times J}$ having the same size is defined by
  (8) $$A*B=\left[\begin{array}{cccc} a_{11}b_{11} & a_{12}b_{12} & \cdots & a_{1J}b_{1J}\\ a_{21}b_{21} & a_{22}b_{22} & \cdots & a_{2J}b_{2J}\\ \vdots & \vdots & \ddots & \vdots \\ a_{I1}b_{I1} & a_{I2}b_{I2} & \cdots & a_{IJ}b_{IJ} \end{array}\right]$$
• Norm
  For a tensor $A\in R^{I_{1}\times I_{2}\times \cdots \times I_{N}}$, its norm can be calculated by
  (9) $$∥A∥=\sqrt{\langle A,A\rangle }=\sqrt{\sum_{i{}_{1}=1}^{I{}_{1}}\sum_{i{}_{2}=1}^{I{}_{2}}\cdots \sum_{i{}_{N}=1}^{I{}_{N}}a{}_{i{}_{1}i{}_{2}\cdots i{}_{N}}^{2}}$$
• Mode-*n* tensor matrix product
  The mode-*n* tensor matrix product of a tensor $A\in R^{I_{1}\times I_{2}\times \cdots \times I_{N}}$ and a matrix $B\in R^{j\times I_{n}}$ is defined by
  (10) $${\left(A\times_{n}B\right)}_{i_{1}\cdots i_{n-1}ji_{n+1}\cdots i_{N}}=\sum_{i_{n}=1}^{I_{n}}a_{i_{1}i_{2}\cdots i_{N}}b_{ji_{n}}$$

### 2.2 Canonical polyadic decomposition

**Fig. 3:**
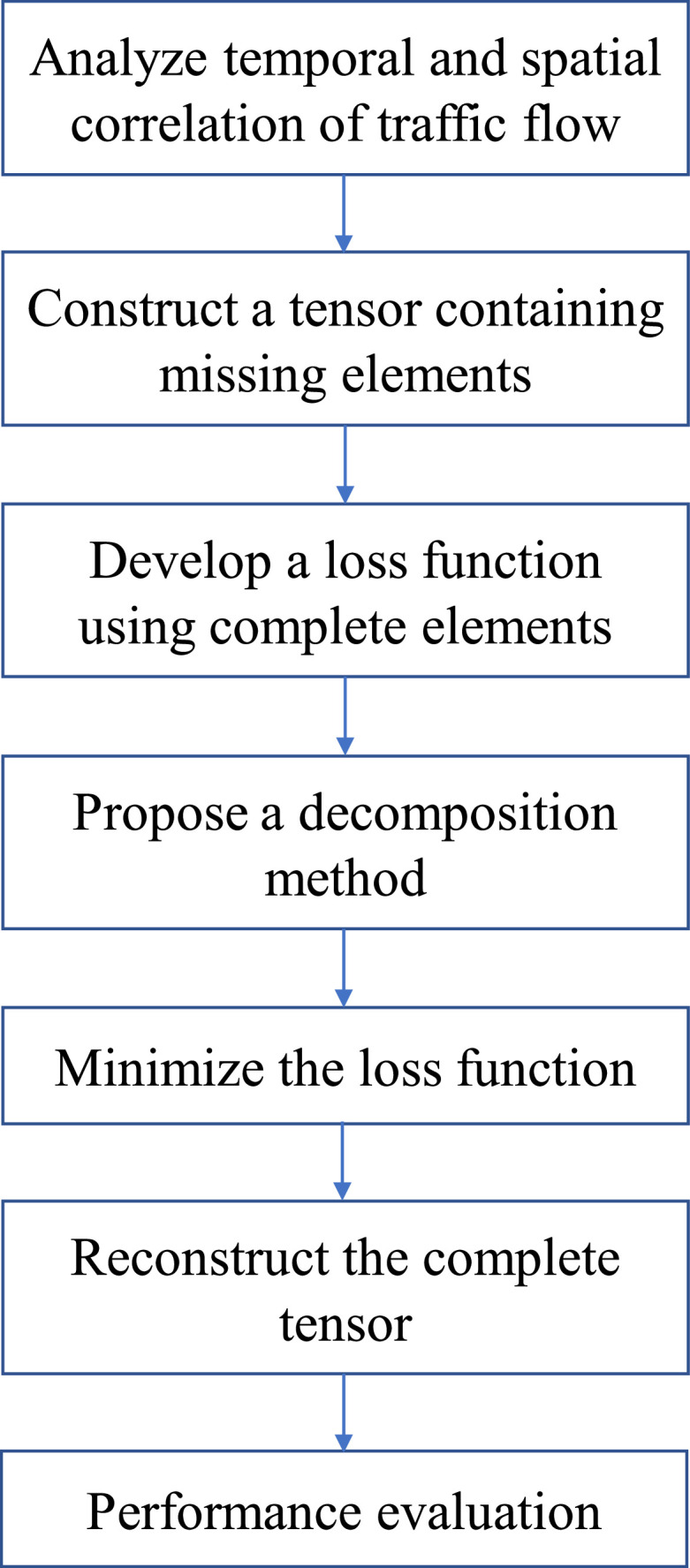
**Procedures of missing data imputation**.

CP decomposition is one of fundamental model for tensor decomposition which is also called as parallel factor analysis, canonical decomposition, and topographic components model. As shown in Fig. 3, the main idea of CP decomposition is to factorize a tensor as the sum of a series of rank-one tensors. By CP decomposition, a *N*th-order tensor, $A\in R^{I_{1}\times I_{2}\times \cdots \times I_{N}}$,M can be written as

(11) $$A\approx \left[\lambda ;A^{\left(1\right)},A^{\left(2\right)},\ldots ,A^{\left(N\right)}\right]d\equiv \sum_{r=1}^{R}\lambda_{r}a_{r}^{\left(1\right)}\circ a_{r}^{\left(2\right)}\circ \cdots \circ a_{r}^{\left(N\right)}$$

where $\lambda \in R^{R}$ and $A^{\left(n\right)}\in R^{I_{n}\times R}$ for n=1,…,N. The above equation can be written in a tensor-matrix product form as

(12) $$A\approx I\times A^{\left(1\right)}\times_{2}A^{\left(2\right)}\cdots \times_{N}A^{\left(N\right)}$$

With the aim to find a decomposition with *R* rank-one tensors to approximate the raw tensor as accurate as possible, take a three-order tensor for example, it can be converted to optimize the following objective function:

(13) $$\min_{\hat{A}}∥A-\hat{A}∥\;\;\text{with}\;\;\hat{A}=\sum_{r=1}^{R}\lambda_{r}a_{r}\circ b_{r}\circ c_{r}$$

The first difficult task to solve the above function is the selection of the number of rank-one tensors *R*. A large number of experiments should be conducted until we find the best *R*. Assuming *R* is fixed, the mission is to compute a decomposition. The most popular algorithm is the alternating least square (ALS) method proposed by Carroll and Chang [3]. By the ALS, $A=[a_{1},a_{2},\ldots ,a_{R}]$, $B=[b_{1},b_{2},\ldots ,b_{R}]$, and $C=[c_{1},c_{2},\ldots ,c_{R}]$ are calculated one by one which means **B** and **C** are fixed when **A** is solved, **A** and **C** are fixed when **B** is solved, and **A** and **B** are fixed when **C** is solved. This procedure continues until a preset criteria is reached. Then, take the solution of **A** for example, if **B** and **C** are fixed, the Eq. 13 can be rewritten as

(14) $$\min_{\hat{A}}{∥X_{\left(1\right)}-\hat{A}{\left(C⊙B\right)}^{⊤}∥}_{F}$$

where $\hat{A}=A\cdot diag\left(\lambda \right)$. Obviously, the problem becomes a least square problem that is easy to solve.

### 2.3 Tucker decomposition

**Fig. 4:**
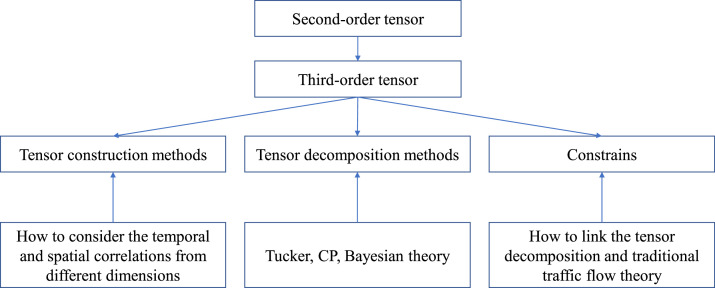
**Summarization of contents in previous studies**.

Tucker decomposition is another popular tensor decomposition method which is called as *N*-mode PCA, *N*-mode SVD, and three-mode factor analysis in previous studies. As shown in Fig. 4, the decomposition of a tensor by Tucker decomposition is similar to the decomposition of a matrix by PCA. Take a three-order tensor for example, it decomposes the tensor into a core tensor multiplied by a matrix along each mode. For a *N*-order tensor, we have

(15) $$A\approx C\times_{1}A^{\left(1\right)}\times_{2}A^{\left(2\right)}\cdots \times_{N}A^{\left(N\right)}$$

where C is the core tensor. Similar to CP decomposition, the objective fnuction of Tucker decomposition for a three-order tensor can be defined as

(16) $$\min_{\hat{A}}∥A-\hat{A}∥\;\;\text{with}\;\;\hat{A}=C\times_{1}A^{\left(1\right)}\times_{2}A^{\left(2\right)}\times_{3}A^{\left(3\right)}$$

In previous studies, some methods have been already been proposed to solve the above function. In this paper, the widely used HOSVD is introduced. Assuming $\hat{A}\in R^{I_{1}\times I_{2}\times \cdots \times I_{N}}$, the Eq. 15 can be rewritten as

(17) $$\begin{array}{c} \min_{C,A^{\left(1\right)},\ldots ,A^{\left(N\right)}}∥A-\left[C\times_{1}A^{\left(1\right)}\times_{2}A^{\left(2\right)}\cdots \times_{N}A^{\left(N\right)}\right]∥\\ \;\text{subject}\;\text{to}\;\hat{C}\in R^{R_{1}\times R_{2}\times \cdots \times R_{N}}\\ A^{\left(n\right)}\in R^{I_{n}\times R_{n}}\;\text{and}\;\text{columnwise}\;\text{orthogonal}\;\text{for}\;n=1,\ldots ,N \end{array}$$

As demonstrated in [2], through a series of derivations, the final objective function becomes

(18) $$∥A^{(n)⊤}W∥\mathrm{with}W=X_{\left(n\right)}\left(A^{\left(N\right)}⊗\cdots ⊗A^{\left(n+1\right)}⊗A^{\left(n-1\right)}⊗\cdots ⊗A^{\left(1\right)}\right)$$

This equation can be solved by SVD by simply setting $A^{\left(n\right)}$ as the $R_{n}$ leading left singular vector of **W**.

### 2.4 Relationship between CP and Tucker decomposition

In fact, CP decomposition can be viewed as a special case of tucker decomposition when the core tensor is super-diagonal and all mode ranks are equal. Moreover, two decomposition are all derived in terms of the sum of some rank-one tensors as shown in Eqs. 12 and 15. Although the form is similar, some differences exist between two decomposition. The first one is the number of components which remains invariant in CP decomposition while can be different in Tucker decomposition. Secondly, from Eqs. 12 and 15, it also can be seen that core tensors of CP and tucker decomposition are different. In the former, the core tensor must be a identity tensor, while in the latter, it can be a any tensor. The final difference is that the solution for CP decomposition is unique with a mild constrain while tucker decomposition is generally not unique without adding some constrains.

## 3 Traffic data imputation

Although a large number of advanced sensors have been installed on highways, missing data problem is still unavoidable in traffic dataset because of the equipment failure, communication outage, and electricity interruption. The existence of missing data brings enormous challenges for all these different transportation applications. For example, the traffic management center needs sufficient real-time traffic data such as traffic flow, traffic speed, and travel time to develop traffic control models. Also, it has been proved the missing data can adversely affect the traffic state prediction models. For example, the accuracy of the deep learning prediction model can be decreased by about 20% by 30% with missing values. Therefore, it is necessary to develop some imputation models for traffic data. In ITS, the traffic data are always collected as a data stream. For the ith sensor, one variable of a series of samples can be constructed as the form of a vector $(v_{1}^{i},\ldots ,v_{N}^{i}$), while D variables should be formed as a matrix:

(19) $$T=\left[\begin{array}{c} V^{1}\\ V^{2}\\ \vdots \\ V^{D} \end{array}\right]=\left[\begin{array}{cccc} v_{1}^{1} & v_{2}^{1} & \cdots & v_{N}^{1}\\ v_{1}^{2} & v_{2}^{2} & \cdots & v_{N}^{2}\\ \vdots & \vdots & \ddots & \vdots \\ v_{1}^{D} & v_{2}^{D} & \ldots & v_{N}^{D} \end{array}\right]$$

where N is the number of samples. When the sampling frequency is set as ten minutes, N=144 samples can be collected per day. After collecting data for T consecutive days, a three-order tensor $A\in R^{D\times N\times T}$ can be obtained (Fig. 2). On highways, a large number of sensors have been installed, and so the traffic data can also be constructed as a four-order tensor. The above examples show that the traffic data can be well expressed by the form of a tensor. In previous studies, different types of tensors have been constructed, and then corresponding methods have been proposed to achieve missing data imputation. The main procedures of missing data imputation are shown in Fig. 3.

### 3.1 Two-order tensor (matrix) imputation

In [4], two consecutive days traffic flow data were used to constructed a two-order tensor, that is a matrix, to capture the fluctuation information. Principle component analysis and some variations were applied to estimate the missing data. The results showed that the probability PCA method was the best one that can improve the root mean square error by at least 25% than some conventional methods. The paper demonstrated that the probability PCA is suitable for application because it does not need some strict assumptions and a large-enough traffic flow dataset. In [5], a different matrix (22×480) was constructed and the performance of probability PCA was tested. The results showed that this method can obtain a satisfactory outcome even when the missing ratio is high (50%). Moreover, this paper proved that the missing ratio in the dataset can negatively affect the traffic prediction model. In [6], a matrix containing both temporal and spatial information was built to estimate missing values. Moreover, it extended the probability PCA and proposed a kernel probability PCA to mine the nonlinear temporal-spatial dependency. The results proved that the incorporating information of neighboring sensors can improve the accuracy. Differently, PCA was applied to impute missing data in a relational dataset to estimate real-time crash likelihood in [7]. This study was a try to decompose the matrix to mine the relationship between traffic flow variables and some external variables. In [8], some widely imputation models were comprehensively compared from accuracy, behaviors, and speed aspects. The results demonstrated probability PCA yielded the best overall performance. Besides, in [9], Bayesian PCA was proposed to imupte the incomplete traffic flow volulme data collected in Beijing. Bayesian PCA is slightly modified from Probability PCA. This algorithm can take a tradeoff among the periodicity, local predictability and also statistical property of the traffic flow. The results showed that the proposed model outperformed the spline interpolation and historical interpolation. In [10], a general linear model based on PPCA was proposed to impute missing not at random traffic speed data. Experimental results showed that the proposed model outperformed the conventional PPCA model. From the above studies, it can be found PCA-based imputation method could consider the daily, weekly, monthly, and spatial correlations of traffic data and construct traffic data into different matrix form to capture these correlations. By utilizing the multiple correlations of traffic data, these methods often outperform traditional time series and interpolation methods.

However, some difficulties still be faced when PCA-based methods were applied. The first one is how to combine the matrix decomposition with some mature theory in the field of transportation. Some researchers have made some tries. For example, in [11], a matrix completion method considering the limitation of road capacity was proposed by adding a constrain to the optimization which could reconstruct a day × interval matrix better even with some outliers. Moreover, the PCA-based methods sometimes were not robust and had the over-fitting problem. To solve this difficulty, an improved low-rank matrix completion (LRMC) method, also called as robust PCA, was proposed in [12]. In [13], LRMC method was combined with K-nearest neighboring in an ensemble framework that can improve the robustness of the method and avoid the over-fitting. Another difficulty is that the matrix only has two dimensions, and so only two modes such as day × hour, day × week, and sensors × week can be considered. Thus, some multi-mode similarities of the traffic data are missed which could result in some poor imputation performance [14]. To overcome this difficulty, some high-order tensors, which can take multi-mode temporal-spatial correlations into consideration, have been constructed and many decomposition method have been proposed to achieve missing data imputation.

Besides there are still some other method based on matrix completion. In [15], the spatial and temporal dependencies were considered as graphs, and a matrix completion algorithm on graphs was proposed. In the context of LRMC, some marginal information prior knowledge of spatial and temporal traffic data is quite important. Xiong et al. added additional constrain condition in low rank completion model in [16]. The proposed algorithm in this paper achieved better performance in both accuracy and speed.

### 3.2 High-order tensor imputation

The first paper applying three-order tensor decomposition in traffic engineering is to estimate missing traffic flow data [14]. In this paper, a 16 days × 24 h × 12 intervals tensor was built to test the randomly missing scenario. The results showed that tucker decomposition outperformed CP decomposition and Bayesian PCA. Especially, the tensor decomposition achieved good performance even when the missing ratio was higher than 50%. Moreover, the paper conducted an experiment to compare the performance of imputation methods when several days’ traffic flow were missing. Unfortunately, matrix-based methods cannot work under this extreme case while tensor-based methods can still work well.

Since then, the multi-mode correlations have been proved in traffic data, and a large number of researchers begin to find the proper decomposition methods. In [17], the performance of least square method, variational Bayesian PCA, fixed point continuation with approximate PCA, and CP decomposition were compared for traffic data imputation in large networks. Differently, it could be found that fixed point continuation with approximate PCA outperformed CP decomposition which indicated that a proper tensor construction and decomposition method could significantly affect accuracy. In [18], the tensor decomposition was seemed as a mixture multi-linear robust PCA optimization problem by adding nuclear norm and L1-norm as constrains and the augmented Lagrange multiplier method was proposed to sovle the parameters. The approach was proved to be more stable and robust. Contrary to add a hard constrain to low the rank of the tensor, softly threshold the core of the decomposition was proposed in [19]. In this paper, authors found the matrix unfolding of the traffic flow tensor exhibited singular values decay fast, and so the tensor was likely to have a compressible core. In order to get a parsimonious representation of a high-order tensor, a single imputation scheme was introduced to compress the core by adding a soft threshold. With real-world dataset, the approach was proved to be accurate and robust. In [20], a four-order tensor (location × time × day × week) was built to finish the traffic flow imputation task. The comparison revealed the consideration of spatial information is important and the increase of spatial information into the tensor could reduce imputation errors.

In [21], some previous tensor-based imputation methods have been reviewed and the tensor train was applied to impute a five-order traffic flow tensor. It found a balanced (higher order) tensor can improve the accuracy of the tensor train factorization and imputation. In [22], a SVD-combined tensor decomposition was proposed to finish traffic speed data recovery. The framework contained three steps: missing data initialization, truncated SVD, and missing data estimation. Benefit from the combination, the hybrid imputation method could consider the ratio threshold of singular values of different tensor unfolding and optimized the size of the core tensor by a sensitivity test. Moreover, the factor matrices from different dimensions were interpreted by visualization. It could be found that the initialization step could suggest a better performance which should be taken into consideration in the future studies.

It can be seen tensors with different orders have been used in previous studies, however few studies focused on how the different representations of traffic data affect accuracy of the imputation. In [23], different forms of tensor including a matrix (214 days × 8784 intervals), a third-order tensor (214 segments × 61 days × 144 intervals), and a fourth-order tensor (214 segments × 9 days × 7days × 144 intervals) were constructed and decomposed by a Bayesian CP decomposition. The paper extended the traditional Bayesian decomposition method to a high-order tensor. The results showed that the third-order tensor outperforms the matrix and the fourth-order tensor and indicated a proper representation is critical but not a higher order. In [24], an iterative step was added into the method in [22] to improve the robustness and evaluated using a traffic flow dataset collected by the automatic number plate recognition system. In order to better characterize the hidden patterns in spatial and temporal traffic data, Chen et al. developed an algorithm under LRTC framework and defined a novel truncated nuclear norm on traffic tensors of location day time of day in [25]. In [26], a Coupled Tensors Completion-Optimization (CTC-OPT) based on a modified Coupled Matrix and Tensor Factorization-Weighted OPTimization algorithm was proposed to impute missing traffic data. The paper organized traffic flow data and speed data into a third-order tensor respectively, and coupled them into road segment mode. And then extracting the common factor matrix from the road segment mode. The results showed that the proposed method outperformed than BCPF, TenALS and BATF. In [27], a traffic speed dataset collected from Guangzhou was constructed into a third-order tensor with a size of 209×61×144, a trafffic flow dataset collected from PeMS was constructed into a third-order tensor with a size of 9×499×288, and a sampling data collected from Madrid M30 highway was organized into a third-order tensor with a size of 311×120×96. And then an improved soft threshold tensor decomposition combining detrending was proposed to impute the above dataset.

Most existing research has not fully utilized the pattern of regular traffic and separated the outliers out from regular traffic. In [28], a robust tensor recovery with fiber outliers model was developed to tackled the mentioned challenge.

Recently, the Bayesian inference incorporated with Markov Chain Monte Carlo methods has draw a great interest in tensor decomposition. A tensor decomposition algorithm combining tensor-train decomposition and Bayesian inference with Markov Chain Monte Carlo Gibbs sampler was proposed to impute missing traffic data [29]. In [30], variational Bayes was introduced into CP decomposition which could automatically learn the parameters of the model. The experiment constructed a third-order tensor (road segment × day × time interval, with a size of 214 × 61 × 144) using the traffic speed data collected from Guangzhou. The results showed that Bayesian augmented tensor factorization (BATF) model could combine explicit patterns and latent factors, and BATF achieved best imputation performance. In [31], a Bayesian nonparametric tensor decomposition (BNPTD) was proposed to impute missing traffic data and discover similarity pattern. In this paper, the smart card data collected from Guangzhou was organized into a third-order tensor(statoin × day × time of the day, with a size of 148 × 14 ×96). Extensive numerical experiment was performed on the constructed tensor, which showed the effectiveness of the proposed model.

Inspired by the coupled matrix-tensor factorization, Jiang et al. developed a missing data imputation algorithm to capture and utilize the spatial and temporal information from heterogeneous data in [32]. In this paper, two auxiliary matrix including a weather feature matrix and a lane feature matrix were coupled in date mode and lane mode of a third-order tensor, respectively. The results showed that auxiliary traffic flow information could effectively improve the imputation performance. In the view of the feature of traffic congestion, an imputation model for traffic congestion data named CIM based on joint matrix factorization was proposed [33]. Compared with existing algorithms that only considered part of traffic congestion features, CIM imputed the missing data with all traffic congestion features including periodicity, temporal coherence and road similarity under the tensor decomposition framework. The results showed that constructing the congestion data into a third-order tensor can capture both temporal periodicity and the similarity between road segments. In previous study, the research on missing data imputation of large-scale traffic data was not sufficient. In [34], researchers utilized urban and temporal information to model spatial and temporal interactions between study regions. More specifically, some additional information related to the urban context of the study area was used to augment the CP algorithm.

Moreover, in [35], a scalable tensor learning model-Low-Tubal-Rank Smoothing Tensor Completion based on the framework of LRTC was proposed. The paper introduced linear transform into large-scale tensor completion problem and equivalently solved the problem through a series of small subproblems. The results showed that the proposed method achieved superior efficiency. The problem of overfitting is very common in the process of large-scale traffic data imputation. In order to tackle this problem, Zhu et al. proposed a Bayesian robust CP decomposition approach [36] that combined the algorithm in [23] and the algorithm in [37]. The approach in this paper not only overcomes the problem of overfitting, but also was robust to the outliers.

Although LRTC has been widely used in previous research, it has some limitations in capturing the local information in complex scenarios. In order to overcome above difficulty, many scholars have carried out some research in such area. In [38], Combining LRTC and sparse self-representation, Chen et al. proposed an algorithm for traffic volume data and traffic speed data. The proposed algorithm benefited from both the advantage of low-rank property and self-similarity characterized by the sparse self-representation. Moreover, in [39], Deng et al. considered the topology of the road network as a graph and utilized graph Fourier transform and t-SVD to process the constructed graph. In [40], the autoregressive time series process was introduced into LRTC to capture both global and local spatial and temporal information in traffic data. In [41], the tensor decomposition model considered the joint imputation of multiple correlative data. Compared with previous studies, the proposed model can learn spatial and temporal non-linear interactions of multiple related tasks,which leaded to better performance.

Optimizing the norm in the loss function is a common way to improve the accuracy of low-rank tensor completion. A trace norm minimizing based tensor completion method is proposed in [42], and resloved by Block Coordinate Descent (BCD) algorithm. Non-convex optimizations based on weighted schatten-p norm minimization was studied in [43]. In this paper, the proposed model consisted of an approximation term and a low-rank regularization term and played a balance role in rank function and nuclear norm. Besides, in [44], the p-shrinkage norm was used to replace tensor rank minimization problem, and the idea of direction weighting was used to solve the dependence of the original model on the data input direction. Tensor nuclear norm also suffers from over-relaxation. In [45], an innovative nonconvex truncated Schatten p-norm for tensors (TSpN) was proposed to approximate tensor rank and impute missing spatial and temporal traffic data.

It can be concluded a large number of tensor-based imputation methods for traffic data, illustrated in Table 1, have been proposed in previous studies from different research perspectives. As shown in Fig. 4, the decomposition method is from second-order to third-order and the content can generally categorized as tensor construction methods, tensor decomposition methods, and constrains.

**Table 1:** **Summarization of tensor decomposition applied for traffic data imputation**.

Paper	Order	Size	Decomposition method	Dataset	Evaluation criteria
[4]	2	288 × 16	Probability PCA	Trffic volume	NMAE, NRMSE, RMSE
[5]	2	22 × 480	Probability PCA	Trffic volume	∖
[6]	2	31 × 288; 92 × 288; 93 × 288;	Kernel PCA	Trffic volume	RMSE
[7]	2	24 × 2674	PCA	Traffic crash	RMSE
[8]	2	43 × 288	Probability PCA	Traffic volume	RMSE
[11]	2	20 × 288	Robust SVD	Traffic volume	MAE, MAPE, SDE
[12]	2	46 × 576	Robust PCA	Traffic volume	RMSE, MAPE
[13]	2	288 × 288	LRMC	Traffic volume	RMSE, MAE
[14]	3	16 × 24 × 12	CP, Tucker	Traffic volume	RMSE
[17]	3	NA	CP	Traffic speed	WRE, Bias, Variance
[18]	3	8 × 7 × 288	Robust PCA	Traffic volume	RSE
[19]	3	68 × 91 × 5760	Extended Tucker	Traffic speed	RMSE, MAPE
[20]	4	11 × 7 × 288 × 2; 11 × 7 × 288 × 3; 11 × 7 × 288 × 5; 11 × 7 × 288 × 7; 11 × 7 × 288 × 9; 11 × 7 × 288 × 11	Tucker	Traffic volume	RMSE
[21]	5	2 × 7 × 16 × 24 × 32	Tensor train	Traffic volume	RSE
[22]	3	61 × 144 × 214	SVD & Tucker	Traffic speed	RMSE, MAE, MRE
[23]	2; 3; 4	214 × 8784; 61 × 144 × 214; 7 × 9 × 144 × 214	Bayesian CP	Traffic speed	MAPE, RMSE
[24]	3	31 × 32 × 237	SVD & Tucker	Traffic volume	MAE, MAPE, RMSE
[26]	3	24 × 60×24	CTC-OPT	Traffic volume & Traffic speed	RSE,TCS
[30]	3	214 × 61×144	BATF	Traffic speed	MAPE, RMSE
[31]	3	148 × 14×96	BNPTD	Traffic volume	MAE, RMSE
[32]	3	5 × 4×288	CMTF	Traffic speed, Traffic volume	MAPE, RMSE
[33]	3	317 × 180×25	CIM	Vehicle passage records	MAE, RMSE, MRE
[34]	3	91 × 91×2880; 1516 × 1516×352	CP	Taxi records	RE
[35]	3	11160 × 28×28; 11160 × 288×56; 35912 × 24×30; 214 × 144×61;	LSTC-Tubal	Traffic speed	MAPE, RMSE
[36]	3	214 × 61×144	BRCP	Traffic speed	MAPE, RMSE
[38]	3	96 × 40×30; 288 × 30×30	LRSSRTC	Traffic volume&Traffic speed	RMSE
[39]	3	288 × 59×307	GT-SVD	Traffic volume	RMSE,MAE,MAPE,$R^{2}$
[40]	3	214 × 144×61; 80 × 108×25; 323 × 288×28; 1156 × 96×31	LATC	Traffic speed	MAPE, RMSE
[41]	3	18 × 28×288	MTNTF	Traffic volume	RMSE,MAE,MAPE
[43]	3	NA	TWSN	Traffic speed	RMSE,MAE,MAPE
[44]	3	214 × 61×144; 323 × 28×288	WLRTC-P	Traffic speed	RMSE,MAPE
[45]	3	144 × 214×61; 96 × 1156×31	LRTC-TSpN	Traffic volume&Traffic speed	RMSE,MAE

MAE: Mean Absolute Error; NMAE: Normalized MAE; RMSE: Root Mean Square Error; NRMSE: Normalized RMSE; MAPE: Mean Absolute Percentage Error; SDE: Standard Deviation of Errors; WRE: Weighted Relative Error; RSE: Relative Square Error TCS: Tensor Completion ScoreMRE: Mean Relative Error

## 4 Traffic state prediction

**Fig. 5:**
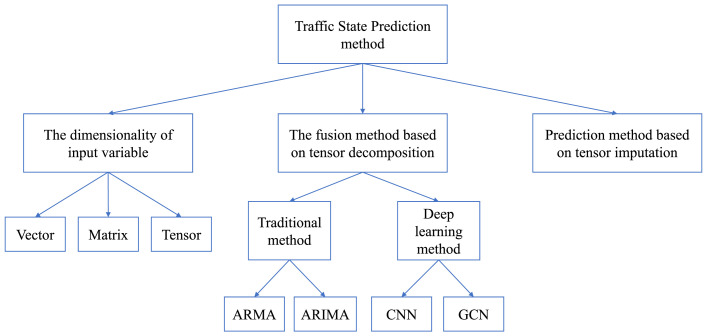
**Traffic prediction method**.

Traffic state prediction aims to estimate traffic flow, travel speed, or travel time of a road segment within a future time window which can contribute to travel route planning and navigation, dynamic traffic control, and other applications. In the formal definition, traffic state prediction task is to predict traffic state $s_{T+1}$ using the observed traffic state information of past time intervals $S=\{s_{t}|t=1,2,\ldots ,T\}$. For multi-step prediction, traffic state at the next n time intervals, ($s_{T+1},\ldots ,s_{T+n}$), can be obtained. The time interval are often different for different applications in practice. According to the length of interval, traffic state prediction can be classified as short-term prediction (5min to 30min), medium-term prediction (30min-60min), and long-term prediction (more than 60min) ([46]). In the following, we will review traffic state prediction task that leverages tensor decomposition and how tensor decomposition methods are applied to improve prediction accuracy. This section contains two parts. We firstly review the studies about tensor tensor prediction, fusion methods of time series prediction and tensor decomposition and then the tensor decomposition-based prediction method as shown in Fig. 5.

### 4.1 Traffic tensor prediction

In recent years, machine learning, especially deep learning, has been witnessed an overwhelming success in transportation engineering. A number of machine learning-based prediction models used tensor as input which were called as traffic tensor prediction in this paper. A reasonable structure of a tensor is important to deep learning models that can help models sufficiently mine the hidden features from the traffic flow data. In the papers about traffic flow prediction just before deep learning models are developed, the one-dimension tensor, that is the vector has been widely used as the input of machine learning models. For example, Makoto et al. compared several machine learning models including random forest, support vector machine, XGBoost, shallow feed-forward neural network, and deep feedforward neural network to test their applicability for short-term traffic prediction in non-recurrent congestion conditions caused by disaster [47]. Li et al. proposed a short-term traffic states forecasting algorithm based on partial least square (PLS) and the traffic state was represented as a vector [48]. With the development of computational devices and emergency of advanced optimization method, the number of layers of neural networks has increased exponentially that can handle high-dimension input. Consequently, researchers have attempt to build high-dimension tensors, containing more correlations, as input of prediction models. For example, Wang et al. transformed the original incomplete matrix to a fourth-order Hankel tensor for traffic state estimation [49]. Ran et al. estimated the traffic state by tucker decomposition from the floating car system. The virtue of high-dimension structure of tensor is that it can take full advantage of the multi-dimensional intrinsic relation of traffic state [50]. In conclusion, tensor pattern, especially high-dimensional tensor pattern, has been the input of the model of machine learning and deep learning extensively with the data explosion and increasing complex temporal-spatial correlations.

### 4.2 Fusion methods of time series prediction and tensor decomposition

Some traditional time series prediction methods based on data statistic techniques such as Autoregressive moving average model (ARMA) and Autoregressive Integrated Moving Average (ARIMA) are applied to estimate traffic state widely. However, most of the traditional prediction models are unable to retain intrinsic features in data as well as their complex temporal-spatial correlations. In recent years, a combination of traditional time series prediction models and tensor decomposition have been a emerging method for traffic states prediction with the development of big data mining and analysis. As a natural big data analyses frame, tensor decomposition not only can keep the spatial and temporal correlation of the raw traffic data, but also can process the big traffic data in high dimension. Therefore, the traditional time series prediction methods are able to generate better results by introducing tensor decomposition. For example, Shi et al. constructed a unified framework incorporating the low-rank block Hankel tensors (BHT) and ARIMA to forecast multiple short time series. Low-rank block Hankel tensors can fully exploit mutual correlations of the historical data by multi-way delay embedding transform (MDT) and ARIMA can predict the future time series precisely based on consecutive core tensors from tucker decomposition. This prediction method was applied a traffic data set for the road occupy rate of Los Angeles County highway network [51]. Li et al. proposed a long-term prediction model combined 2D-ARMA with CP decomposition, which can update prediction in real-time in an effective way. This model was applied to predict the real passenger flow data in Hong Kong URT system successfully [52].

By taking advantage of the combination of traditional time series prediction methods and tensor decomposition, the accuracy and efficiency of traffic state prediction will be improved effectively and the diversity of the traditional methods can be further expansion. In [53], researchers combined the CP-WOPT and diffusion convolution gated recurrent unit (DCGRU) models to forecast the traffic condition. An experiment conducted on the ride-hailing service data collected from Nanjing, and the results showed that the method in this paper outperformed other traditional models. In previous study, traffic flow prediction suffered from the data sparsity problem. To tackle such issue, Yang et al. proposed tensor based algorithm to model traffic flows, which could capture the spatial and temporal pattern of traffic flow and did not need pre-training [54]. In [55], Liu et al. developed a two-step prediction algorithm based on tensor completion framework. To be specifically, the algorithm could estimate the missing data and decompose the traffic data into different components simultaneously. Compared with conventional three-step strategy, two-step strategy could significantly reduced the risk of amplifying the approximation error. Then, the Prophet model and a Gaussian process regression model could be used to predict the components, respectively. Moreover, to address the high uncertainty of the traffic flow system and the multimode correlation of traffic flow data, the grey prediction model of tensor decomposition algorithm was proposed by organically combining the modelling mechanism of the grey prediction model and the theory of the tensor decomposition algorithm [56]. In [57], Tucker decomposition least squares algorithm was used to establish the tensor alternating least squares GM (1,1) model by combining the modelling mechanism of the grey classical model GM (1,1) with Tucker decomposition. The results showed that its effect was far better than the other five benchmark grey prediction models.

To predict the traffic flow, Yan et al. proposed a spatial and temporal missing data imputation algorithm based on Residual Value Tensor Decomposition (MDCA-RVTD), which combined linear regression, univariate spline, and CP decomposition [58]. Then the prediction problem could be transformed into a missing data imputation problem and the algorithm mentioned above could predict the future traffic flow. In [59], CP decomposition was used to capture the temporal feature of entry-traffic flows. Then, the future temporal features could be forecasted by ARIMA model which could reflect the temporal characteristic of future entry-traffic flows. Finally, tensor decomposition was implemented to obtain the future entry traffic flows.

With the rapid development of deep learning, a lot of novel algorithm based on artificial neural network (ANN), a data-driven model without prerequisites, have been exploited to predict the traffic state and made an excellent achievement. Like the combination mentioned above, a combination of ANN and tensor decomposition is a creative method for traffic states prediction. Many literatures have been demonstrated the superiority of the integrated methods of convolutional neural network (CNN) and tensor decomposition. For example, Han et al. developed a multi-dimensional learning machine for predicting the traffic speed which combined a CNN with tensor decomposition. Tensor decomposition based methods mainly consider temporal-spatial neighbourhood element while CNN can train the prediction model from historical experience [60]. Xu et al. proposed a factorized spatial-temporal tensor graph convolutional network (GCN) to predict traffic speed. Tucker decomposition was apply to derive factorized a tensor convolution, which performs separate filtering in small-scale space, time, and feature modes to reduce the computational burden and suppress noise [61]. Chen et al. proposed a tensor-train fuzzy deep convolution (TFDC) approach for citywide traffic flow prediction.

Tucker decomposition was introduced into the TFDC model to compress parameters for traffic flow feature learning with high prediction accuracy and low computational burden [62]. Wu et al. focused on proposing a series of tensor-based RNNs (T-RNNs) and a T-RNNs based multi-modal prediction approach (TMMP) to provide accurate prediction services. Firstly, three types of T-RNNs including tensor-based vanilla RNN, tensor-based long short-term memory (T-LSTM) and tensor-based gated recurrent unit (T-GRU) were proposed, in which the input, output and weights were arbitrary high-order tensors. Then, to compress the weight parameters, we further proposed two compact TT-based GRU (TT-GRU) and Tucker based GRU (Tucker-GRU) models by applying tensor train(TT) and Tucker decompositions to T-GRU model. Afterwards, based on the high-order output tensor generated by T-RNNs, a TMMP approach was proposed to achieve the accurate predictions under various scenarios [63]. Based on the graph representation, the tensor decomposition combined temporal similarity revisited graph convolutional gate recurrent unit (T-TRGCGR),ingeniously combining traffic prediction and data completion through the Graph Laplace, was proposed to predict traffic states under partially input data missing circumstances and sparse detector distribution for a large-scale freeway network [64].

In conclusion, most of deep learning methods need a mass of computing resources and time. The tensor decomposition such as tucker decomposition can generate the core tensor which is smaller than the raw data and noise suppression. Therefore, the combination of deep learning method and tensor decomposition can achieve state-of-the-art performance with high efficiency.

### 4.3 Tensor decomposition-based prediction method

**Fig. 6:**
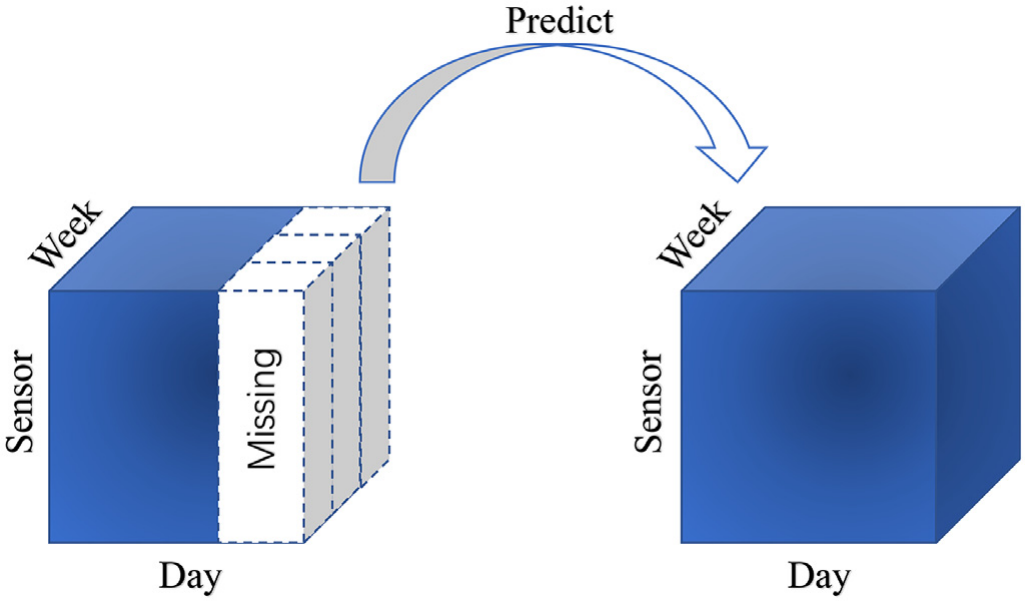
**The situation of fiber-like missing**.

Tensor decomposition-based imputation method has been proved that have high-performance for processing incomplete traffic data. The task of traffic state prediction can transfer to fill the fiber-like missing entries locating in the end of time series. As shown in Fig. 6, we can fill the blank area (missing entries) from the information of dark area (intact entries) by tensor decomposition-based imputation method. Moreover, tensor decomposition-based method can achieve outstanding prediction results with even the incomplete data sets. For example, Chen et al. propose a Bayesian temporal tensor factorization (BTTF) framework for both imputation and forecasting from large-scale and multidimensional spatio-temporal data sets (including traffic data sets). The forecasting model can achieve real-time prediction by multi-step rolling prediction scheme based on imputation model [65]. Tan et al. proposed a short-term traffic flow prediction method based on dynamic tensor completion (DTC) that the traffic data are indicated as a dynamic tensor pattern [66]. In brief, tensor decomposition-based prediction method usually implemented based on the imputation method.

This model develops tensor formed dynamic mode decomposition, recording the dynamic information of traffic data into a state transition tensor. In addition, the model took low rank property of the dynamic tensor and the similarity of temporal variation trend into consideration [67].

## 5 Analysis of travel pattern

Origin-destination (OD) data plays an important role in fundamental traffic engineering filed such as transport planning, operations and management to promote the efficiency of urban transportation system [68]. For the road traffic system, OD data can provide the command staff some satisfactory solutions for traffic congestion. For rail transit system, it also brings accurate and appropriate train scheduling and train route allocation. The significant objectives of travel pattern prediction include improving urban mobility, promoting residents trip degree of satisfaction and lifting efficiency of intersection region. The task of analysis the travel pattern can be transformed into the understanding the OD data from a multi-dimension perspective.

With the development of information collecting equipment and high-performance computers, the massive and multi-way OD data can be collected from ubiquitous sensors which changes the structure of traditional data sources of urban mobility patterns. The traditional OD data usually can be obtained from family questionnaire survey which have some obvious limitations such as the issue of privacy and memory [69]. The real-time movement trajectory data of pedestrians and vehicles can be collected from GPS signals, Bluetooth devices and the records of public transport smart card transactions, which provide researchers a new opportunity and a reliable way to gain insight into the mobility patterns of smart cities [[70], [71], [72], [73]]. However, the organizational form and analytical method of the plentiful and complex urban mobility data are thorny problem, which have been increasing concerned research directions. The extracting of key features and mining spatio-temporal correlations from the urban mobility data is the essential content about the understanding of urban mobility patterns.

**Fig. 7:**
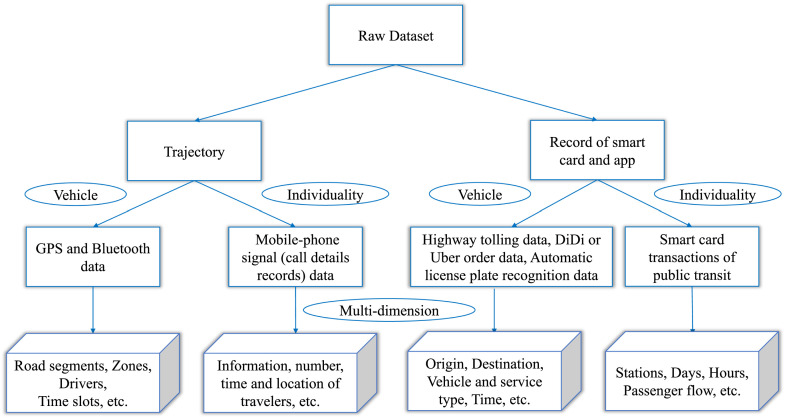
**The organization of OD tensor from the raw dataset**.

The analysis methods for traditional OD data usually base on two-dimension matrix and rely on prerequisite assumptions for the models, which has a lot of intrinsic limitation. The OD data can be organized into the high-dimension form easily and naturally because of the spatio-temporal correlation and regularity of daily track of people and the multiple source of the data. As a natural multi-dimensional data analysis tools and data-driven method, tensor decomposition has been successfully applied in diverse scientific filed such as signal processing image reconstruction and machine learning [74]. The applications of tensor decomposition for processing OD data mainly including prediction, denosing and imputation by the low rank tensor approximation algorithms. Moreover, tensor decomposition has been a primary method for discovering the spatial and temporal patterns of human mobility and understanding the urban regional dynamic from the record of public transport smart card, vehicle GPS data and traffic flow data. The organizational structure of OD tensor from the raw dataset will be shown in Fig. 7 Based on the pattern of OD tensor, there are a lot of study for urban dynamic and travel pattern. For example, Qi et al. proposed a regional mobility patterns analysis and prediction model which integrates the inner-restricted fuzzy C-means clustering, tucker decomposition and artificial neural network using smart card data and points of interest (POI) data [75]. Han et al. achieved significant results on clustering and prediction of temporal evolution of global congestion configurations in a large-scale urban transportation network based on the non-negative tensor factorization [76]. In [77], Tucker decomposition was used to discover spatial clusters, temporal patterns, and the relationships between the inbound ridership in the Barcelona metro. In recent years, The non-negative tensor decomposition technique has been utilized to extract the features of traffic flow [[78], [79]]. Tang et al. applied Non-negative CP decomposition to uncover the characteristics of travel patterns from temporal and spatial dimensions in the metro network of Shenzhen City [80]. A fourth-order tensor was constructed to describe the spatial and temporal characteristics of people various mobility motifs. Non-negative tensor decomposition was used to identify the principal patterns of time, space, and daily motifs in a city and the level of interactions between them[81]. Besides, in [82], Wang et al. also utilized a Non-negative Tucker decomposition to identify the city-scale human mobility pattern and extensive experiment was conducted on massive mobile phone signal data. Moreover, Cao et al. utilized Non-negative Tucker decomposition to analyze the types of travel patterns based on the origins and destinations in bike-sharing data [83]. In [84], Tensor robust principal component analysis was applied for the purpose of discovering distinctive normal and abnormal traffic patterns. The performance of the tensor-based algorithm usually better than traditional matrix-based method for the prediction and imputation thanks to the consideration of the multi-dimension correlations of data simultaneously. Through the better and novel results, researchers can understand and reveal the latent structures of travel pattern in urban area from the brand-new perspectives. For example, discovering the law of administrative division and evolution of city from the pattern of population mobility and hunting for a fluent transportation network by diagnosing traffic-disease. More researches on tensor decomposition for OD data will be shown in Table 2.

**Table 2:** **Summarization of tensor decomposition applied for travel pattern**.

Paper	Area	Decomposition method	Dataset	Application
[85]	Guangdong province, China	CP decomposition	The highway tolling data	Forecast future traffic demand
[86]	Ishikawa prefecture, Japan	CP decomposition	Mobile-phone location data	Solving the difficulties in observing long-distance travel behavior and clearly extracting groups with different elasticities
[87]	Beijing, China	CP decomposition	GPS trajectories received from taxicabs	Citywide travel time estimation; To estimate the travel times of any road segment in a road network under different traffic conditions in different time slots
[88]	NYC, USA and THS, Greece	CP decomposition	GPS trajectories	Traffic volume prediction by low rank approximations of the original tensor considering the traffic reciprocity at different pair of locations.
[89]	Hong Kong	CP decomposition	Public transport smart card transactions in metro stations	Imputation for incomplete data; Prediction of passenger flow in metro stations
[31]	Guangzhou, China	CP decomposition	Public transport smart card transactions in metro stations	Achieving incomplete traffic data imputation and similarity pattern discovery simultaneously
[90]	Xian, China	CP decomposition	OD matrix data from Urban rail transit including traffic passenger volumes	Imputation for incomplete data
[91]	Brisbane, Australia	Tucker decomposition	Bus passenger trajectory obtained from smart card data and Bluetooth trajectory data	Extracting underlying spatio-temporal movement patterns of city
[92]	Singapore	Tucker decomposition	Public transport smart card transactions including both bus and metro modes	Reconstruction of the joint distribution using a low-rank approximation and simple latent structures to reveal the spatial- temporal patterns of urban mobility
[93]	Beijing, China	Tucker decomposition	Taxi GPS trajectories and POI records	Urban dynamics discovery; City-disease diagnosing and urban planning
[94]	Beijing, China	Tucker decomposition	Public transport smart card transactions including both bus and metro modes	Identification meaningful citywide transit patterns and citywide transit hot spots by an OTD tensor
[95]	Changsha, China	Tucker decomposition	The traffic flow from four straight lines	Imputation for incomplete data; Prediction of the traffic flow tensor
[96]	Hangzhou, China	Tucker decomposition	Millions of trips from mobile phone signaling data on the city scale for one week in AM and PM peak hours in Hangzhou	Revealing the spatial-temporal patterns of urban mobility
[97]	Hangzhou, China	Tucker decomposition	DiDi order data and land use GIS data	Solving classification problems in mobility pattern analysis and interpreting and understanding passengers dynamic ridesharing patterns
[98]	Shenzhen, China	Tucker decomposition	GPS trajectories received from the sampled vehicle	To estimate cycle-based traffic volume at signalized intersections

Although tensor decomposition for OD data have been widely applied, there are some deserve discussion of limitation. Firstly, the previous studies usually focus on offline data but the trajectory data of GPS and mobile-phone signal is real-time and sequential. So, some online algorithm for tensor decomposition should be exploited. Secondly, a uniform way to self-adapting finding the optimal core tensor size for tensor decomposition should be developed. Thirdly, due to the high-dimensional structure of OD tensor and the curse of dimensionality, the computing time and memory resources for the tensor decomposition-based algorithms usually are a heavy burden. It is necessary to reduce the time and space complexity of the algorithms.

## 6 Other tasks

Anomaly detection is a vital task in ITS. Detecting these anomalies in spatial and temporal traffic activities can provide practical insights for traffic monitoring and operation. In previous study, the widely used anomaly detection model built on dynamic linear models including time-varying autoregressive and switching Kalman filtering/smoothing. To detect anomalies accurately, the tensor-based algorithms have been proposed to address such issue. In [99], Wang et al. focued on detecting anomalies in spatial and temporal traffic demand data and proposed an algorithm combined Tucker probabilistic tensor decomposition and spectral-based techniques. Extensive experiments were conducted in the traffic simulation software SUMO. Besides, in order to tackled the scalability issue in traffic big data and the issue of measuring anomalys effect from the complex spatial and temporal dependencies, Wang et al. developed a low rank dynamic tensor learning model to identify anomalous [100]. Besides, non-negative CP decomposition was used to detect anomalies in traffic tensor constructed by speed transition matrix [101]. In [102], Graph Regularized Low-rank plus Temporally Smooth Sparse decomposition was proposed for spatial and temporal anomaly detection, which took the spatially sparse and temporally smooth structure of urban anomalies into account.

Moreover, tensor decomposition model has been extended to nonrecurrent traffic congestion detection and traffic objects location estimation. For example, in [103], Li et al. proposed a coupled scalable Bayesian robust tensor factorization model to detect nonrecurrent traffic congestion. The proposed model could recognize the spatial and temporal patterns from multivariate traffic data including traffic volume, traffic speed and road density which could be used to detect nonrecurrent traffic congestion. In terms of traffic objects location estimation in ITS, Guo et al utilized CP decomposition to process transportation big data to improve the real-time performance of location estimation [104].

## 7 Discussions and insights

In this section, a summary on the applicability of tensor decomposition in ITS is provided. In addition, the advantages and shortcomings of the tensor decomposition is presented. Finally, the tricks for the tensor decomposition in specific applications are addressed.

### 7.1 Applicability

From the review, it can be seen that tensor decomposition is mainly applied in three fundamental tasks: traffic data imputation, traffic state prediction, and analysis of travel pattern. The aim of the former two tasks are similar that using tensor decomposition to mine the temporal and spatial correlations in the traffic flow data, and then the correlations would be used to reconstruct the raw data to achieve imputation or prediction. For the third task, the temporal and spatial correlation among the massive travel data can be extracted to explain the travel pattern of the citizen. Among the application, the former two tasks are mainly to improve the accuracy of the proposed algorithms for tensor decomposition, while the last task is mainly to break down a high-dimensional tensor into lower-dimensional components and explain each component. On the one hand, tensor decomposition can be used in supervised learning problems of transportation engineering, where the goal is to predict a target variable based on input features. On the other hand, tensor decomposition is a powerful tool for discovering meaningful patterns in complex, multi-dimensional data without the need for explicit supervision.

### 7.2 Advantages of tensor decomposition

• **Interpretability.** Tensor decomposition can be used to reduce the dimensionality of a high-dimensional transportation temporal and spatial dataset, making it easier to analyze and visualize the data. Then, the results by identifying the underlying factors contribute to the observed patterns in the traffic flow. This can be useful in applications such as traffic data imputation and traffic flow prediction, where understanding the underlying factors can help to improve the accuracy of the algorithms.
• **Data efficiency.** Tensor decomposition is data-efficient meaning it requires less data to achieve good performance. This can be useful in transportation applications such as traffic incident detection where collecting large amounts of data is difficult or expensive. Thus, the data efficiency of tensor decomposition makes it a promising tool in cases where the number of available samples is limited.
• **Unsupervised ability.** Tensor decomposition is especially well-suited for unsupervised learning tasks involving travel pattern analysis where the relationships between traveling variables are not well-understood. By modeling the urban temporal and spatial data as a tensor and decomposing it into lower-dimensional components, tensor decomposition can identify patterns and relationships that might be difficult to detect using other unsupervised learning techniques.
• **Compression.** Tensor decomposition can reduce the storage requirements of high-dimensional temporal and spatial data by representing it in a more compact form. Moreover, by choosing an appropriate decomposition method and tuning the parameters, it is often possible to achieve significant compression without sacrificing too much accuracy. This can be particularly useful in transportation applications where a large volume data would be generated each day among the city-level road network.

### 7.3 Shortcomings of tensor decomposition

• **Linear model.** Traditional tensor decomposition, such as Tucker decomposition and CP decomposition, assume that the relationships between the modes of tensor are linear. However, many real-world transportation temporal and spatial datasets exhibit nonlinear relationships, which cannot be captured by linear models.
• **Organization.** Tensor decomposition may not be suitable for handling sequential data such as time-series data and unstructured data. Organizing the tensor in a way that takes into account the different types of data can be challenging, and may require specialized techniques such as feature engineering or data normalization. Moreover, tensors can have a large number of dimensions and it can be challenging to identify meaningful patterns or relationships in high-dimensional temporal and spatial data. Therefore, organizing the tensor in a way that makes sense for the analysis can be a complex task.
• **Selection of ranks.** The selection of ranks for tensor decomposition is a critical step in the process, as it determines the number of components or factors that will be extracted from the tensor. The choice of ranks can have a significant impact on the quality and interpretability of the results, however, expert knowledge of the domain or the data were used to select the optimal ranks in some cases and we still face challenges for ranks selection.
• **Computational complexity.** The decomposition algorithm such as the Higher Order Orthogonal Iteration (HOOI) algorithm, have a higher computational complexity but are more accurate for some types of tensors, while others, such as the Alternating Least Squares (ALS) algorithm, are faster but may not converge to the optimal solution.

### 7.4 General guideline for tensor design and application

In this review, we have covered various transportation-related applications that use tensor decomposition. Generally speaking, tensor decomposition can solve both supervised problems and unsupervised problems. Overall, the design of tensors in transportation engineering requires careful consideration of variables, dimensionality, loss function, and optimization.

• **Consideration of variables.** The first step in designing tensors for transportation engineering is to identify the variables of interest. These can include traffic volume, speed, travel time, vehicle type, road geometry, weather conditions, and other relevant factors.
• **Dimensionality.** Once the variables have been identified, the next step is to determine the dimensionality of the tensors. In transportation engineering, tensors can be one-dimensional (e.g., representing a single road segment), two-dimensional (e.g., representing a road network), or higher-dimensional (e.g., representing multiple modes of transportation).
• **Loss function.** For supervised problems, the loss functions should be designed. Common loss functions include Mean Squared Error (MSE), Root Mean Squared Error (RMSE), and Kullback-Leibler Divergence (KL divergence). The choice of loss function depends on the nature of the data and the objective of the tensor decomposition.
• **Optimization.** For supervised problems, an proper optimization algorithm, such as gradient descent, should be selected to minimize the chosen loss function. The optimized tensor decomposition model will provide a reconstructed tensor that minimizes the reconstruction error. For unsupervised problems, the traditional decomposition methods can be directly applied.

## 8 Conclusion and future directions

In this review, we provide a comprehensive survey of how tensor decomposition is applied in various transportation applications using temporal and spatial data. In particular, four types of tasks have been intensively examined, including traffic data imputation, traffic state prediction, analysis of travel pattern, and other tasks. The first two applications are always defined as supervised problems and the entire line of literature develops some new methods based on tensor decomposition to solve them. The third application is always defined as unsupervised problems and the aim is to find the essential patterns among massive temporal and spatial data. Also, we review some other tasks. We also discuss the advantages and shortcomings of tensor decomposition to handle temporal and spatial data.

In the following, we also summarize the future directions of further combination between tensor decomposition and temporal and spatial data in transportation engineering from different directions.

Direction 1: classification problems in transportation-related application. From the review, it can be seen tensor decomposition have been widely applied in regression problems. A classification problem in transportation-related application can involve predicting a particular outcome or category based on a set of input features which could be extracted by tensor decomposition. Some possible classification tasks in transportation could include predicting the type of transportation mode, identifying the severity of traffic accidents, and predicting the likelihood of a particular route.

Direction 2: image processing in traffic engineering. Tensor decomposition has been widely used to analyze and extract information from multi-dimensional data, such as images and videos. Image processing plays a critical role in traffic engineering by providing valuable insights and information about traffic conditions. In the future research, the application of tensor decomposition in transportation-related image processing could be paid more attention.

Direction 3: physical-informed tensor decomposition. Physical knowledge and constraints containing in temporal and spatial data can provide valuable prior knowledge to tensor decomposition, which can help guide the learning process and improve the interpretability of the results. These benefits make physical-informed tensor decomposition an important research area with many potential applications in transportation field.

Direction 4: nonlinear tensor decomposition of transportation temporal and spatial data. Most existing tensor decomposition techniques are linear and cannot capture nonlinear relationships in the traffic flow data. Nonlinear tensor decomposition is essential for capturing complex and nonlinear relationships in transportation system and should be paid more attention in the future studies.

## Declaration of competing interest

The authors declare that they have no conflicts of interest in this work.

## References

[1] Kiers H.A. (2000). Towards a standardized notation and terminology in multiway analysis. J.Chemom. J. Chemom. Soc..

[2] Kolda T.G., Bader B.W. (2009). Tensor decompositions and applications. SIAM Rev..

[3] Carroll J.D., Chang J.J. (1970). Analysis of individual differences in multidimensional scaling via an n-way generalization of “Eckart-Young” decomposition. Psychometrika.

[4] Qu L., Li L., Zhang Y. (2009). PPCA-based missing data imputation for traffic flow volume: A systematical approach. IEEE Trans. Intell. Transp. Syst..

[5] Chen C., Wang Y., Li L. (2012). The retrieval of intra-day trend and its influence on traffic prediction. Transp. Res. Part C Emerg. Technol..

[6] Li L., Li Y., Li Z. (2013). Efficient missing data imputing for traffic flow by considering temporal and spatial dependence. Transp. Res. Part C Emerg. Technol..

[7] Ke J., Zhang S., Yang H. (2019). PCA-based missing information imputation for real-time crash likelihood prediction under imbalanced data. Transp. A Transp. Sci..

[8] Li Y., Li Z., Li L. (2014). Missing traffic data: Comparison of imputation methods. IET Intel. Transport Syst..

[9] Qu L., Zhang Y., Hu J. (2008). 2008 IEEE Intelligent Vehicles Symposium.

[10] Huang L., Li Z., Luo R. (2022). Missing traffic data imputation with a linear generative model based on probabilistic principal component analysis. Sensors.

[11] Tan H., Wu Y., Cheng B. (2014). Robust missing traffic flow imputation considering nonnegativity and road capacity. Math. Prob. Eng..

[12] Luo X., Meng X., Gan W. (2019). Traffic data imputation algorithm based on improved low-rank matrix decomposition. J. Sens..

[13] Chen X., Wei Z., Li Z. (2017). Ensemble correlation-based low-rank matrix completion with applications to traffic data imputation. Knowl. Based Syst.

[14] Tan H., Feng G., Feng J. (2013). A tensor-based method for missing traffic data completion. Transp. Res. Part C Emerg. Technol..

[15] Han T., Wada K., Oguchi T. (2019). 2019 IEEE Intelligent Transportation Systems Conference - ITSC.

[16] Xiong Z., Wei Y., Xu R. (2022). Low-rank traffic matrix completion with marginal information. J. Comput. Appl. Math..

[17] Asif M.T., Mitrovic N., Dauwels J. (2016). Matrix and tensor based methods for missing data estimation in large traffic networks. IEEE Trans. Intell. Transp. Syst..

[18] Tan H., Cheng B., Feng J. (2014). Mixture augmented lagrange multiplier method for tensor recovery and its applications. Discrete Dyn. Nat. Soc..

[19] Goulart J.d.M., Kibangou A., Favier G. (2017). Traffic data imputation via tensor completion based on soft thresholding of tucker core. Transp. Res. Part C Emerg. Technol..

[20] Ran B., Tan H., Wu Y. (2016). Tensor based missing traffic data completion with spatial–temporal correlation. Physica A.

[21] Pastor G. (2018). A low-rank tensor model for imputation of missing vehicular traffic volume. IEEE Trans. Veh. Technol..

[22] Chen X., He Z., Wang J. (2018). Spatial-temporal traffic speed patterns discovery and incomplete data recovery via SVD-combined tensor decomposition. Transp. Res. Part C Emerg. Technol..

[23] Chen X., He Z., Sun L. (2019). A bayesian tensor decomposition approach for spatiotemporal traffic data imputation. Transp. Res. Part C Emerg. Technol..

[24] Zhang H., Chen P., Zheng J. (2019). Missing data detection and imputation for urban ANPR system using an iterative tensor decomposition approach. Transp. Res. Part C Emerg. Technol..

[25] Chen X., Yang J., Sun L. (2020). A nonconvex low-rank tensor completion model for spatiotemporal traffic data imputation. Transp. Res. Part C Emerg. Technol..

[26] Zhou W., Zheng H., Feng X. (2019). 2019 11th International Conference on Wireless Communications and Signal Processing (WCSP).

[27] Gong C., Zhang Y. (2020). Urban traffic data imputation with detrending and tensor decomposition. IEEE Access.

[28] Hu Y., Work D.B. (2020). Robust tensor recovery with fiber outliers for traffic events. ACM Trans. Knowl. Discov. Data (TKDD).

[29] Salehi H. (2021). Nondestructive Characterization and Monitoring of Advanced Materials, Aerospace, Civil Infrastructure, and Transportation XV.

[30] Chen X., He Z., Chen Y. (2019). Missing traffic data imputation and pattern discovery with a bayesian augmented tensor factorization model. Transp. Res. Part C Emerg. Technol..

[31] Han Y., He Z. (2020). Simultaneous incomplete traffic data imputation and similarity pattern discovery with bayesian nonparametric tensor decomposition. J. Adv. Transp..

[32] Jiang H., Deng H. (2021). Expressway traffic flow missing data repair method based on coupled matrix-tensor factorizations. Math. Prob. Eng..

[33] Jia X., Dong X., Chen M. (2021). Missing data imputation for traffic congestion data based on joint matrix factorization. Knowl. Based Syst..

[34] Said A.B., Erradi A. (2021). Spatiotemporal tensor completion for improved urban traffic imputation. IEEE Trans. Intell. Transp. Syst..

[35] Chen X., Chen Y., Saunier N. (2021). Scalable low-rank tensor learning for spatiotemporal traffic data imputation. Transp. Res. Part C Emerg. Technol..

[36] Zhu Y., Wang W., Yu G. (2022). A bayesian robust CP decomposition approach for missing traffic data imputation. Multimed. Tools Appl..

[37] Zhao Q., Zhou G., Zhang L. (2015). Bayesian robust tensor factorization for incomplete multiway data. IEEE Trans. Neural Netw. Learn. Syst..

[38] Chen X., Liang S., Zhang Z. (2022). A novel spatiotemporal data low-rank imputation approach for traffic sensor network. IEEE Internet Things J..

[39] Deng L., Liu X.-Y., Zheng H. (2021). Graph spectral regularized tensor completion for traffic data imputation. IEEE Trans. Intell. Transp. Syst..

[40] Chen X., Lei M., Saunier N. (2021). Low-rank autoregressive tensor completion for spatiotemporal traffic data imputation. IEEE Trans. Intell. Transp. Syst..

[41] Zhu Y., Wang J., Wang J. (2022). Multitask neural tensor factorization for road traffic speed-volume correlation pattern learning and joint imputation. IEEE Trans. Intell. Transp. Syst..

[42] Lin Y., Li Q., Guo D. (2021). Tensor completion-based trajectory imputation approach in air traffic control. Aerosp. Sci. Technol..

[43] Wang S., Zhao Y., Zhang Y. (2022). Spatiotemporal traffic data imputation via tensorial weighted schatten-p norm minimization. IET Intel. Transport Syst..

[44] Wu J., Zhao Y., Zhang H. (2022). Spatio-temporal traffic data tensor restoration method based on direction weighting and p-shrinkage norm. Math. Prob. Eng..

[45] Nie T., Qin G., Sun J. (2022). Truncated tensor schatten p-norm based approach for spatiotemporal traffic data imputation with complicated missing patterns. Transp. Res. Part C Emerg. Technol..

[46] Yu H., Wu Z., Wang S. (2017). Spatiotemporal recurrent convolutional networks for traffic prediction in transportation networks. Sensors.

[47] Chikaraishi M., Garg P., Varghese V. (2020). On the possibility of short-term traffic prediction during disaster with machine learning approaches: An exploratory analysis. Transp. Policy (Oxf).

[48] Li W., Wang J., Fan R. (2020). Short-term traffic state prediction from latent structures: Accuracy vs. efficiency. Transp. Res. Part C Emerg. Technol..

[49] X. Wang, Y. Wu, D. Zhuang, et al. Low-rank Hankel tensor completion for traffic speed estimation, arXiv preprint arXiv:2105.11335(2021).

[50] Ran B., Song L., Zhang J. (2016). Using tensor completion method to achieving better coverage of traffic state estimation from sparse floating car data. PLoS ONE.

[51] Shi Q., Yin J., Cai J. (2020). Proceedings of the AAAI Conference on Artificial Intelligence.

[52] Li Z., Yan H., Zhang C. (2020). Long-short term spatiotemporal tensor prediction for passenger flow profile. IEEE Rob. Autom. Lett..

[53] Jia R., Li Z., Xia Y. (2020). Urban road traffic condition forecasting based on sparse ride-hailing service data. IET Intel. Transport Syst..

[54] Yang F., Liu G., Huang L. (2020). Tensor decomposition for spatial-temporal traffic flow prediction with sparse data. Sensors.

[55] Liu C., Wu T., Li Z. (2021). Individual traffic prediction in cellular networks based on tensor completion. Int. J. Commun. Syst..

[56] Tong M., Duan H., Luo X. (2021). Research on short-term traffic flow prediction based on the tensor decomposition algorithm. J. Intell. Fuzzy Syst..

[57] Duan H., Xiao X., Long J. (2020). Tensor alternating least squares grey model and its application to short-term traffic flows. Appl. Soft Comput..

[58] Yan J., Li H., Bai Y. (2021). Spatial-temporal traffic flow data restoration and prediction method based on the tensor decomposition. Appl. Sci..

[59] Gao H., Wang Z., Yan Z. (2021). Synchronized entry-traffic flow prediction for regional expressway system based on multidimensional tensor. Transp. Res. Rec..

[60] Han T., Tang K., Oguchi T. (2020). Short-term travel speed prediction for urban expressways using convolutional neural network and tensor decomposition. Transp. Res. Procedia.

[61] X. Xu, T. Zhang, C. Xu, et al. Spatial-temporal tensor graph convolutional network for traffic prediction, arXiv preprint arXiv:2103.06126(2021).

[62] Chen W., An J., Li R. (2019). Tensor-train fuzzy deep computation model for citywide traffic flow prediction. IEEE Access.

[63] Wu Q., Jiang Z., Hong K. (2021). Tensor-based recurrent neural network and multi-modal prediction with its applications in traffic network management. IEEE Trans. Netw. Serv. Manage..

[64] Dong H., Ding F., Tan H. (2022). Laplacian integration of graph convolutional network with tensor completion for traffic prediction with missing data in inter-city highway network. Physica A.

[65] Chen X., Sun L. (2021). Bayesian temporal factorization for multidimensional time series prediction. IEEE Trans. Pattern Anal. Mach. Intell..

[66] Tan H., Wu Y., Shen B. (2016). Short-term traffic prediction based on dynamic tensor completion. IEEE Trans. Intell. Transp. Syst..

[67] Zhang X., Zhang Y., Wei X. (2022). Traffic forecasting with missing data via low rank dynamic mode decomposition of tensor. IET Intel. Transport Syst..

[68] Krishnakumari P., van Lint H., Djukic T. (2019). A data driven method for od matrix estimation. Transp. Res. Procedia.

[69] Madre J.-L., Axhausen K.W., Brög W. (2007). Immobility in travel diary surveys. Transportation (Amst).

[70] Batty M., Axhausen K.W., Giannotti F. (2012). Smart cities of the future. Eur. Phys. J. Spec. Top..

[71] Gonzalez M.C., Hidalgo C.A., Barabasi A.-L. (2008). Understanding individual human mobility patterns. Nature.

[72] Roth C., Kang S.M., Batty M. (2011). Structure of urban movements: Polycentric activity and entangled hierarchical flows. PLoS ONE.

[73] Sun L., Jin J.G., Axhausen K.W. (2015). Quantifying long-term evolution of intra-urban spatial interactions. J. R. Soc. Interface.

[74] A. Cichocki, N. Lee, I.V. Oseledets, et al. Low-rank tensor networks for dimensionality reduction and large-scale optimization problems: Perspectives and challenges Part 1, arXiv preprint arXiv:1609.00893(2016).

[75] Qi G., Huang A., Guan W. (2018). Analysis and prediction of regional mobility patterns of bus travellers using smart card data and points of interest data. IEEE Trans. Intell. Transp. Syst..

[76] Han Y., Moutarde F. (2016). Analysis of large-scale traffic dynamics in an urban transportation network using non-negative tensor factorization. Int. J. Intell. Transp. Syst. Res..

[77] Frutos-Bernal E., Martín del Rey Á., Mariñas-Collado I. (2022). An analysis of travel patterns in barcelona metro using tucker3 decomposition. Mathematics.

[78] Yang S., Wu J., Xu Y. (2019). Revealing heterogeneous spatiotemporal traffic flow patterns of urban road network via tensor decomposition-based clustering approach. Physica A.

[79] Zhong H., Qi G., Guan W. (2019). Application of non-negative tensor factorization for airport flight delay pattern recognition. IEEE Access.

[80] Tang J., Wang X., Zong F. (2020). Uncovering spatio-temporal travel patterns using a tensor-based model from metro smart card data in Shenzhen, China. Sustainability.

[81] Shi S., Wang L., Wang X. (2022). Uncovering the spatiotemporal motif patterns in urban mobility networks by non-negative tensor decomposition. Physica A.

[82] Wang D., Cai Z., Cui Y. (2022). Nonnegative tensor decomposition for urban mobility analysis and applications with mobile phone data. Transp. A Transp. Sci..

[83] Cao M., Huang M., Ma S. (2020). Analysis of the spatiotemporal riding modes of dockless shared bicycles based on tensor decomposition. Int. J. Geogr. Inf. Sci..

[84] Lykov S., Asakura Y. (2020). Anomalous traffic pattern detection in large urban areas: Tensor-based approach with continuum modeling of traffic flow. Int. J. Intell. Transp. Syst. Res..

[85] Ren J., Xie Q. (2017). 2017 18th IEEE International Conference on Mobile Data Management (MDM).

[86] Yamaguchi H., Nakayama S. (2020). Detection of base travel groups with different sensitivities to new high-speed rail services: Non-negative tensor decomposition approach. Transp. Policy (Oxf).

[87] Tang K., Chen S., Liu Z. (2018). Citywide spatial-temporal travel time estimation using big and sparse trajectories. IEEE Trans. Intell. Transp. Syst..

[88] Bhanu M., Mendes-Moreira J., Chandra J. (2021). Embedding traffic network characteristics using tensor for improved traffic prediction. IEEE Trans. Intell. Transp. Syst..

[89] Li Z., Sergin N.D., Yan H. (2020). Proceedings of the AAAI Conference on Artificial Intelligence.

[90] Dong H., Ding F., Tan H. (2021). Rail transit od-matrix completion via manifold regularized tensor factorisation. IET Intel. Transp. Syst..

[91] Naveh K.S., Kim J. (2018). Urban trajectory analytics: Day-of-week movement pattern mining using tensor factorization. IEEE Trans. Intell. Transp. Syst..

[92] Sun L., Axhausen K.W. (2016). Understanding urban mobility patterns with a probabilistic tensor factorization framework. Transp. Res. Part B Methodol..

[93] Wang J., Wu J., Wang Z. (2019). Understanding urban dynamics via context-aware tensor factorization with neighboring regularization. IEEE Trans. Knowl. Data Eng..

[94] Du B., Zhou W., Liu C. (2019). Transit pattern detection using tensor factorization. INFORMS J. Comput..

[95] Duan H., Liu Y., Wang D. (2019). Prediction of a multi-mode coupling model based on traffic flow tensor data. J. Intell. Fuzzy Syst..

[96] Wang D., Cai Z., Cui Y. (2019). Nonnegative tensor decomposition for urban mobility analysis and applications with mobile phone data. Transp. A Transp. Sci..

[97] Zhu Z., Sun L., Chen X. (2021). Integrating probabilistic tensor factorization with bayesian supervised learning for dynamic ridesharing pattern analysis. Transp. Res. Part C Emerg. Technol..

[98] Tang K., Tan C., Cao Y. (2020). A tensor decomposition method for cycle-based traffic volume estimation using sampled vehicle trajectories. Transp. Res. Part C Emerg. Technol..

[99] Wang X., Fagette A., Sartelet P. (2019). 2019 IEEE Intelligent Transportation Systems Conference (ITSC).

[100] Wang X., Sun L. (2021). Diagnosing spatiotemporal traffic anomalies with low-rank tensor autoregression. IEEE Trans. Intell. Transp. Syst..

[101] Tišljarić L., Fernandes S., Carić T. (2021). Spatiotemporal road traffic anomaly detection: Atensor-based approach. Appl. Sci..

[102] Sofuoglu S.E., Aviyente S. (2022). Gloss: tensor-based anomaly detection in spatiotemporal urban traffic data. Signal Process..

[103] Li Q., Tan H., Jiang Z. (2021). Nonrecurrent traffic congestion detection with a coupled scalable bayesian robust tensor factorization model. Neurocomputing.

[104] Guo Y., Wang X., Lan X. (2022). Traffic target location estimation based on tensor decomposition in intelligent transportation system. IEEE Trans. Intell. Transp. Syst..

